# The *X* Gene of Adeno-Associated Virus 2 (AAV2) Is Involved in Viral DNA Replication

**DOI:** 10.1371/journal.pone.0104596

**Published:** 2014-08-15

**Authors:** Maohua Cao, Hong You, Paul L. Hermonat

**Affiliations:** 1 Central Arkansas Veterans Healthcare System, Little Rock, Arkansas, United States of America; 2 Department of Obstetrics and Gynecology, University of Arkansas for Medical Sciences, Little Rock, Arkansas, United States of America; National Institutes of Health, United States of America

## Abstract

Adeno-associated virus (AAV) (type 2) is a popular human gene therapy vector with a long active transgene expression period and no reported vector-induced adverse reactions. Yet the basic molecular biology of this virus has not been fully addressed. One potential gene at the far 3′ end of the AAV2 genome, previously referred to as *X* (nt 3929 to 4393), overlapping the 3′ end of the *cap* gene, has never been characterized, although we did previously identify a promoter just up-stream (p81). Computer analysis suggested that *X* was involved in replication and transcription. The X protein was identified during active AAV2 replication using a polyclonal antibody against a peptide starting at amino acid 98. Reagents for the study of *X* included an AAV2 deletion mutant (dl78-91), a triple nucleotide substitution mutant that destroys all three 5′ AUG-initiation products of *X*, with no effect on the *cap* coding sequence, and *X*-positive-293 cell lines. Here, we found that *X* up-regulated AAV2 DNA replication in differentiating keratinocytes (without helper virus, autonomous replication) and in various forms of 293 cell-based assays with help from wild type adenovirus type 5 (wt Ad5) or Ad5 helper plasmid (pHelper). The strongest contribution by *X* was seen in increasing wt AAV2 DNA replication in keratinocytes and dl78-91 in Ad5-infected *X*-positive-293 cell lines (both having multi-fold effects). Mutating the *X* gene in pAAV-RC (pAAV-RC-3Xneg) yielded approximately a ∼33% reduction in recombinant AAV vector DNA replication and virion production, but a larger effect was seen when using this same X-knockout AAV helper plasmid in *X*-positive-293 cell lines versus normal 293 cells (again, multi-fold). Taken together these data strongly suggest that AAV2 *X* encodes a protein involved in the AAV life cycle, particularly in increasing AAV2 DNA replication, and suggests that further studies are warranted.

## Introduction

First utilized in 1984 [1]–[4], adeno-associated virus (AAV) (type 2) is rapidly growing in popularity as a preferred gene therapy vector with a long transgene delivery period and high safety record [5]–[7]. From the sequencing of adeno-associated virus type 2 (AAV2) in 1983 and the phenotypic study of AAV mutants, there have been three *trans* phenotypes identified within the AAV2 genome [1], [8]. The *rep* phenotype, defective for DNA replication and transcription, encodes replication/transcription factor proteins Rep78, Rep68, Rep52, and Rep40. Another *trans* phenotype discovered is *lip* (described as *inf* by Barrie Carter's group) [1], [8] which produces viral particles of low infectivity (missing VP1). The third phenotype discovered is the *cap* genotype which doesn't produce any viral particles at all (encoding the major structural protein, VP3). Just recently, a fourth *trans* phenotype, the *AAP* gene, involved in virion maturation, has been identified by the Kleinschmidt group [9].

However, the molecular biology of AAV is not completely understood in the rush to develop gene therapy products and protocols from it. To highlight this deficit, the gene which we originally referred to as “*X*” gene, at the far 3′ end of AAV2 (1999) has never been characterized [10]. The *X* open reading frame, at the extreme 3′ end of the genome from nt 3929 to 4393 (map units 84–94), is contiguous with the carboxy-terminus of the VPs, but in another reading frame [10], [11]. This open reading frame (ORF) has been ignored and has changed only slightly over time as sequence corrections have been made.

Three lines of evidence strongly suggest that *X* is an actual gene. First, its length (155 aa) statistically favors (>99%) that *X* is a real gene, encoding a real protein. Second, we have previously identified an AAV promoter, p81, just upstream of the *X* open reading frame (ORF), indicating it has a dedicated promoter, and strongly suggesting that p81-*X* is a natural transcriptional cassette within AAV2 [10]. Both reverse transcription-primer extension and S1 nuclease protection analysis were used to identify this promoter. Third, most isolates of AAV type 2 confirm the presence of the *X* open reading frames positioned in the vicinity of the 3′ end of the *cap* gene with regions.

In search of a phenotype for the *X* gene, and guided by computer predictions of the likely function(s) of X, we investigated whether it may have a function in AAV DNA replication. Here we find that *X* has a significant effect on autonomous AAV DNA replication in differentiating normal human skin cells. Additionally, *X* also has a significant, moderate to minor, effect on full length AAV2 genomes as well as recombinant (r) DNA replication and virion production using the standard pAAV-RC helper plasmid in HEK293 cells.

## Results

### Computer analysis and generation of *X* reagents

**Figure 1:**
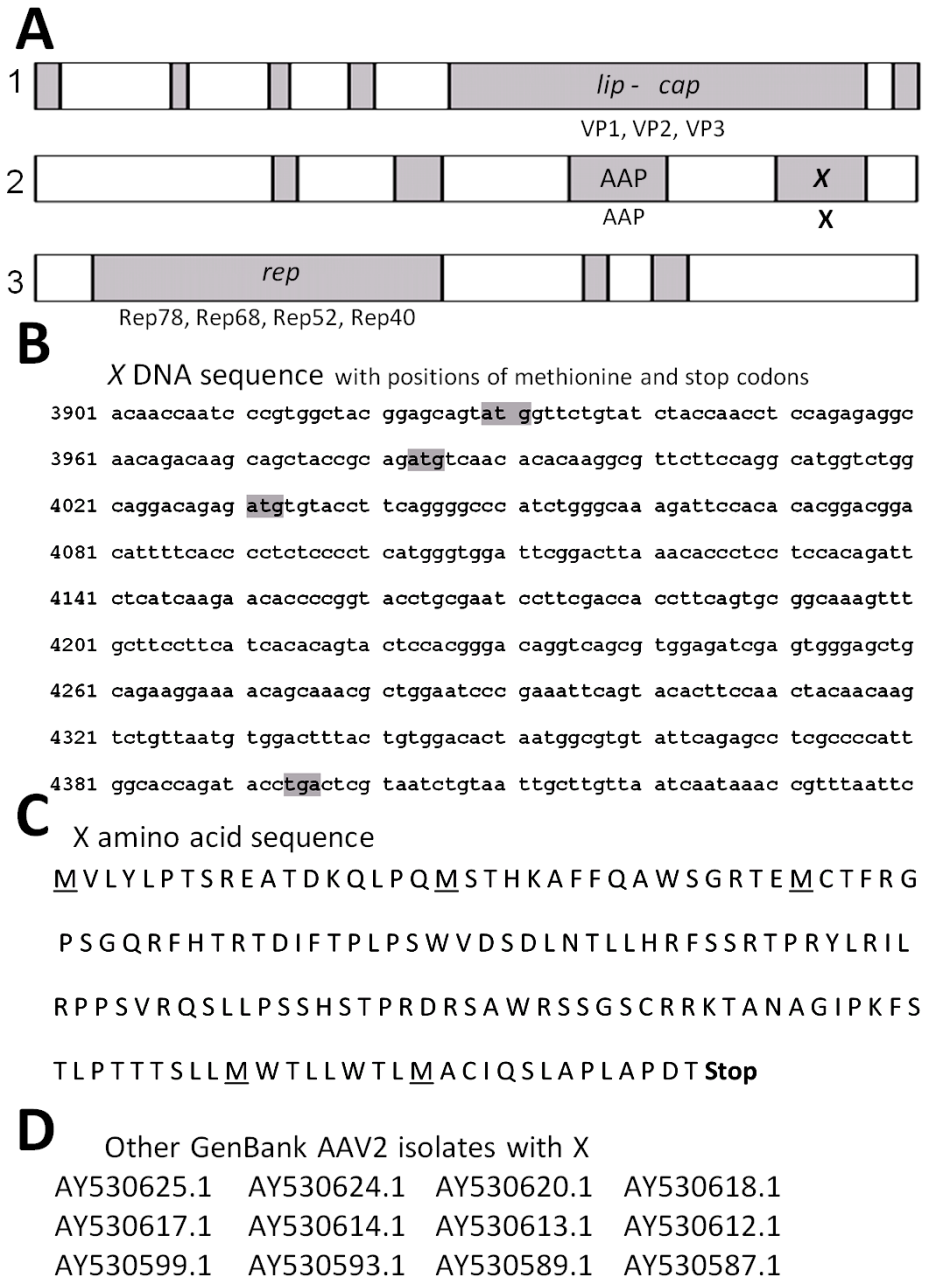
Sequences of AAV2 X. **A** shows the open reading frames (ORF), reading from the natural AAV2 promoters (left to right), as analyzed by NIH ORF finder software analysis of NC_001401 (AAV2, Kleinschmidt) with their names/functions indicated at the top of the figure as determined by mutational analysis [1]. **B** shows the nucleotide (nt) sequence of the third largest ORF, called *X* [10], of AAV2 with its start methionines and stop codon highlighted in grey. **C** shows the amino acid (aa) sequence of the *X*. **D** shows a series of AAV2 isolates found in Genbank which also show the *X* ORF.

**Figure 2:**
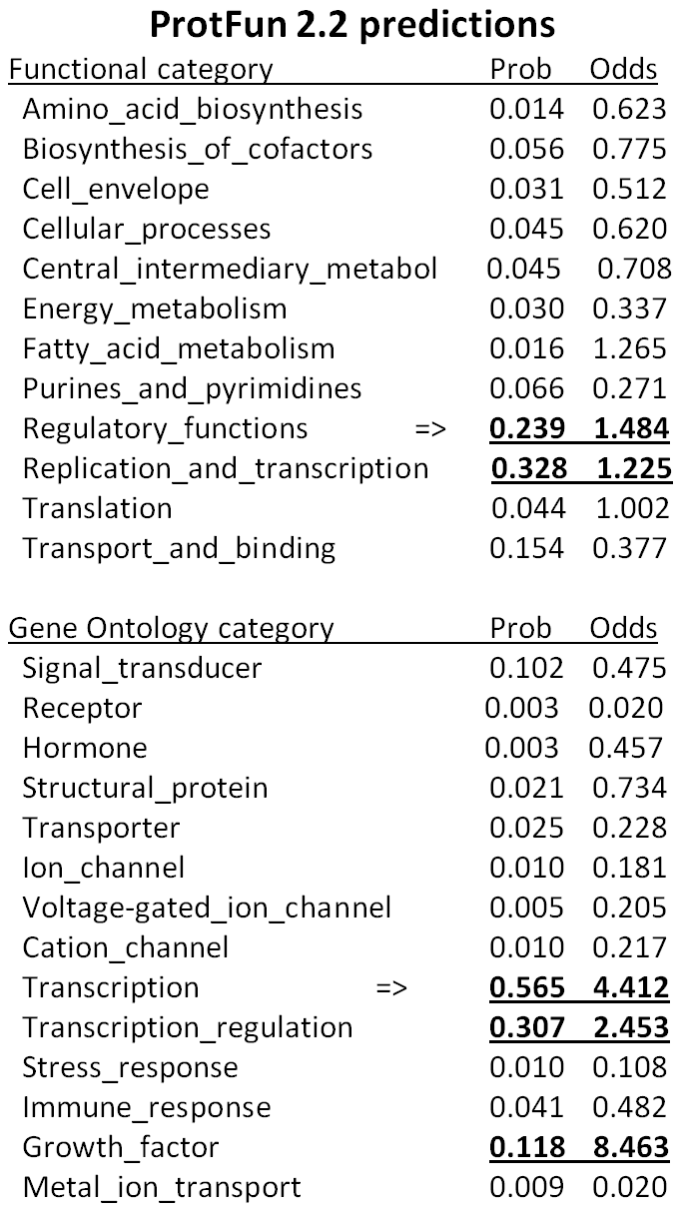
Software predicted functions of *X*. **A** shown are results from Technical University of Denmark, ProtFun 2.2 software analysis which attempts to predict functions of proteins. Notice that replication and transcription are listed multiple times.

**Figure 3:**
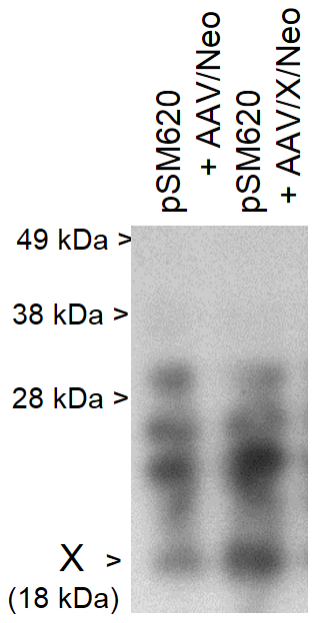
Identification of X protein. Shown is a Western blot of protein from 293 cells infected with Ad5 and transfected with pSM620 (wt AAV2) plus either AAV/Neo or AAV/X/Neo. The Western blot was probed with polyclonal rabbit antibodies directed against a peptide derived from aa 98–111of AAV2 *X*. While polyclonal antibodies are well known for having cross-reactivity, note that a protein of approximately 18 kDa, the predicted size of X, is seen strongly enhanced in cells transfected with AAV/X/Neo, consistent with X.

X is a rather significant ORF of 465 base pairs, 155 amino acids. Figure 1A, shows a cartoon of the AAV2 genome and includes the relative position of genes/open reading frames (ORF). Figure 1B shows the DNA sequence of the AAV2 *X* ORF derived from NCBI Reference Sequence NC_001401.2, and Figure 1C shows the corresponding amino acid sequence derived from the *X* ORF. Figure 1D shows other AAV2 isolates that also contain the X ORF. Moreover, ProtFun2.2 software analysis (Technical University of Denmark, Center for Biological Sequence Analysis), shown in Figure 2, suggests functions of transcription and replication, and growth factor for *X*. ProtFun2.2 software analysis is based on functional attributes of associated amino acids in a sequence, post-translational modifications, likely trafficking, and basic parameters such as length and isoelectric point. Because of the results of this computer analysis we analyzed whether we might be able to identify an actual X protein. Figure 3 shows a Western blot of protein from Ad5-infected 293 cells, with pSM620 (wt AAV2) co-transfected with AAV/Neo or AAV/X/Neo, using the anti-98-X polyclonal antibody, which identifies an enhanced protein band at the correct size for X (X is estimated to be ∼18 kDa) when AAV/X/Neo is present, but is also present at a lower level in the control lane. A second antibody, generated against another X-derived peptide, gave a similar result (data not shown).

**Figure 4:**
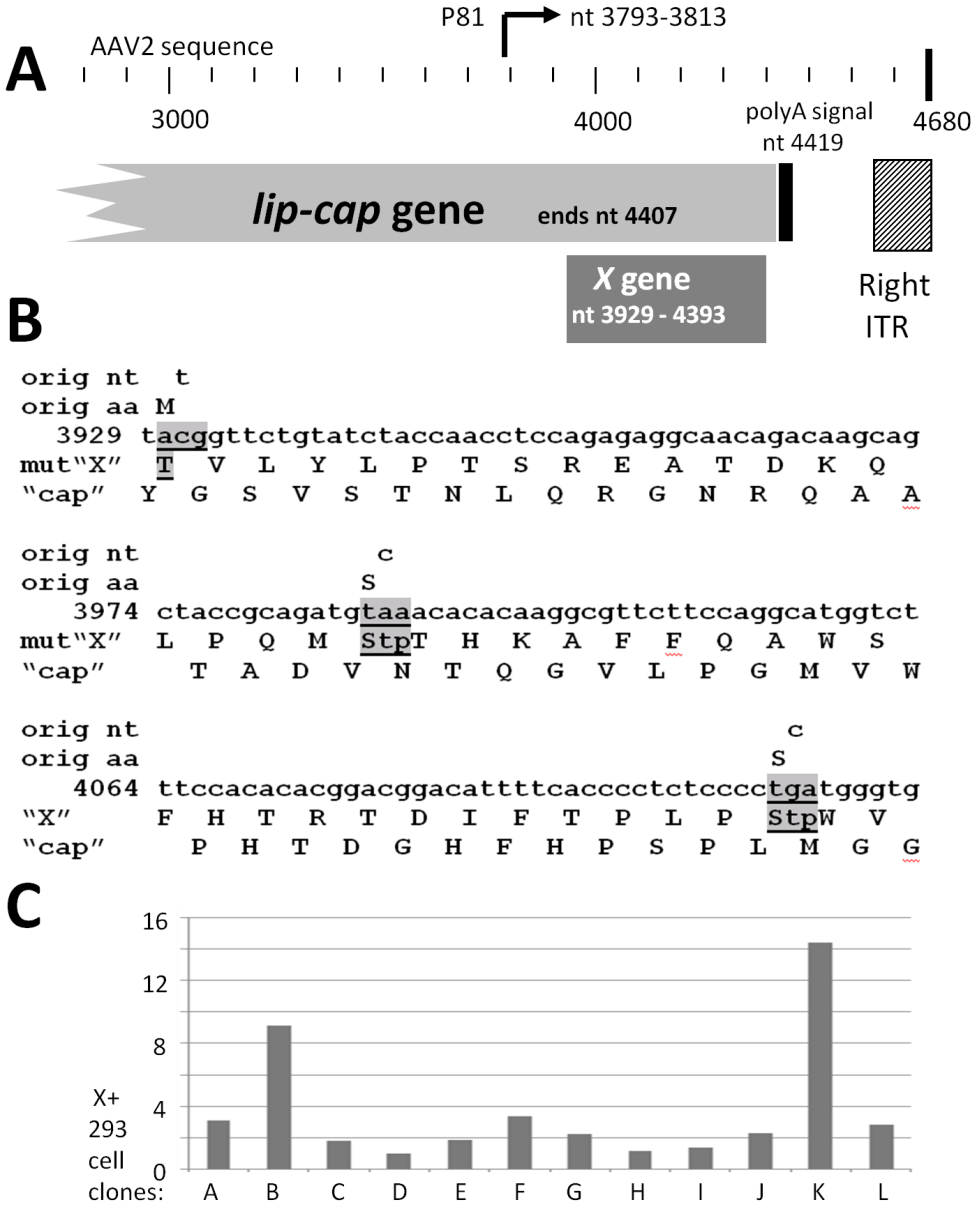
Environs of the *X* gene and reagents for X study. **A** shows the region of *X* at the 3′ end of AAV2. Included are the 3′ end of *lip-cap* [1], [11], the p81 promoter [10], the poly A sequence and the 3′, right, inverted terminal repeat (ITR). **B** shows three nt substitution mutations in *X* which eliminate the products from all three 5′/amino end X start methionines, but which have no effect on the *cap* ORF/coding sequence. **C** shows the analysis of twelve 293 cell clones generated by transfection of pCI/*X*/Neo, and then G418 selected. The left scale shows the copy number of *X* found by Q-RT-PCR with clone D as the “1X” reference clone. 293-X-B and 293-X-K, having the highest copy numbers of *X* were chosen for further study.

We then generated multiple types of *X-*related constructs (Figure 4). The vicinity of *X* within the AAV2 genome is shown in Figure 4A. One mutant we constructed (Figure 4B) is a triple knockout of the *X* ORF without any change in the coding of the *cap* gene. That is, VP1, VP2 and VP3 remain unaltered, while all products from the three 5′ start methionines of the *X* ORF are eliminated. This triple mutant section was inserted into both pAAV-RC and pSM620 to give pAAV-RC-3Xneg and pSM629-3Xneg, respectively. A series of 293 cell lines carrying the *X* gene was generated by transfecting with pCI/*X*/Neo and then carrying out G418 selection. A number of these 293-X-Neo resistant cell lines were analyzed by Q-PCR to determine the copy number of *X* which they carried within (Figure 4C). 293-X clones B and K were chosen for further study as they had the highest *X* copy number among the 12 clones analyzed.

### *X* contributes to autonomous AAV2 replication in skin rafts

**Figure 5:**
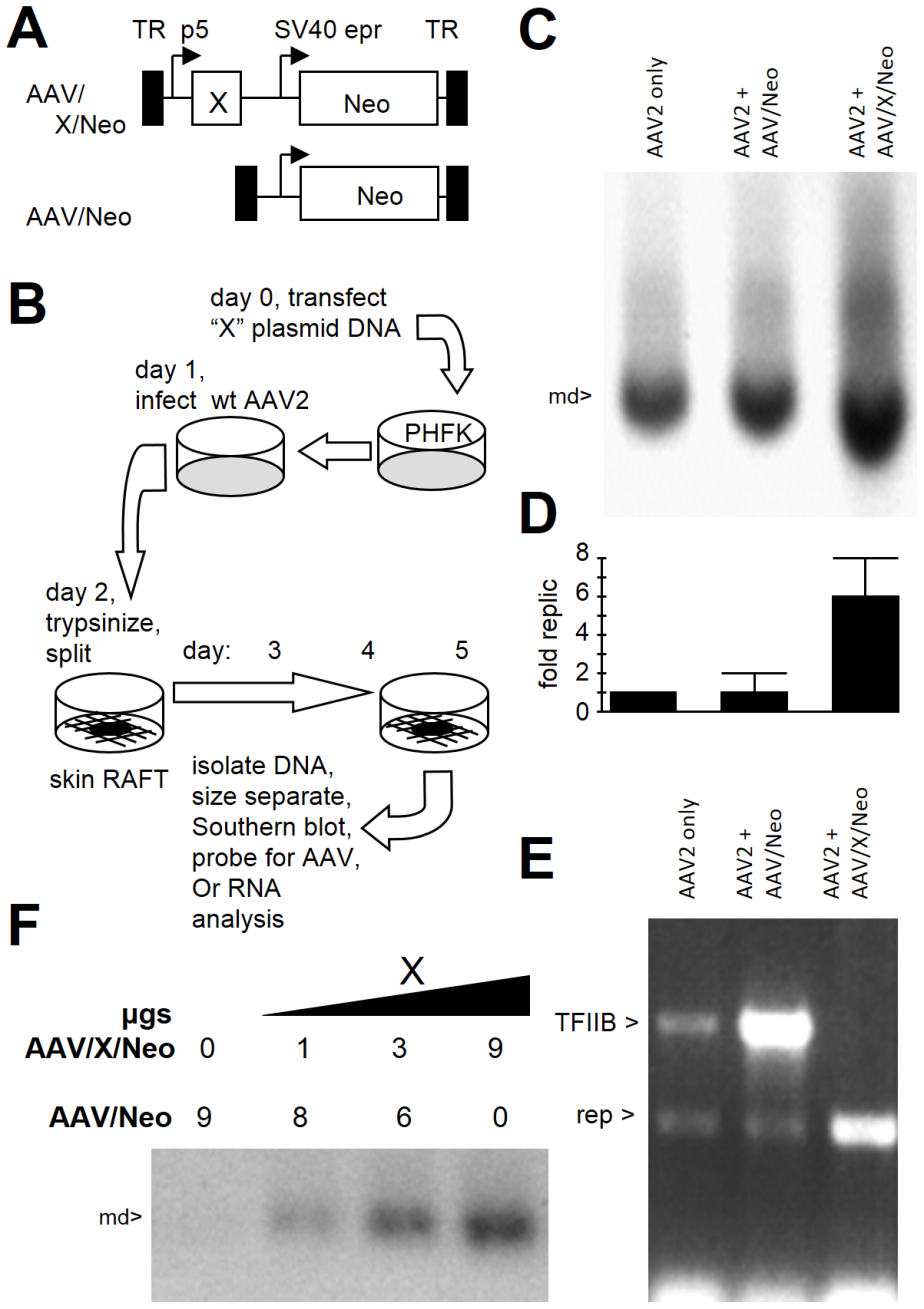
*X* enhances AAV2 autonomous DNA replication in skin rafts. **A** shows the structure of the AAV vector plasmids used. **B** shows the structure of the experiment analyzing *X* gene function in the skin raft (stratified squamous epithelium, autonomous AAV2 replication). Note that the plasmid is transfected before infection of the keratinocytes with wt AAV. This is done so as to allow the transfected gene to be expressed during the early phase of wt AAV replication. **C** shows the resulting Southern blot of DNA after probing the membrane with 32P-cap sequences (but not including X sequences). **D** shows a quantification of five such experiments. Note that AAV2 DNA replication is enhanced 6 fold. **E** shows that *X* enhances AAV2 *rep* expression relative to housekeeping TFIIB gene expression. These data are fully consistent with the higher DNA replication found in **C. F** shows dosage dependent affect of adding X. Note that the larger the amount of AAV/X/Neo transfected the higher the level of AAV2 DNA replication.

*X* may be involved in wild type AAV2's natural host tissue, stratified squamous epithelium, such as that found in the nasopharynx or genital tract, known to harbor AAV2. Additionally AAV2 is known to autonomously replicate in differentiating skin cells [12]–[15]. We therefore utilized the organotypic epithelial raft culture system (skin raft) to analyze effects of *X* on AAV2 autonomous DNA replication. Primary human foreskin keratinocytes (PHFK) were transfected with X expressing plasmid (Figure 5A), or control Neo only plasmid, as shown in Figures 5B, and then these cells were subsequently infected with wild type AAV2. No adeno-, herpes-, or papilloma-virus helper virus were used in this experiment. The next day these cells were used to generate a skin raft as shown in Figure 5B. After day 5 of keratinocyte stratification (skin development) the skin rafts were harvested and analyzed for both DNA replication and *rep* gene RNA expression. Figure 5C shows a ^32^P-cap DNA probed Southern blot of a representative gel (of three total done). As can be seen the level of monomer duplex (md) AAV2 DNA (4.7 kb) is approximately six fold higher in the presence of *X* plasmid transfection when analyzed by densitometric quantification of the autoradiograph shown in Figure 5D. A reverse transcriptase polymerase chain reaction (RT-PCR) analysis of *rep* RNA expression was done and Figure 5E shows that the ratio of *rep* to TF_II_B housekeeping control gene was highest in the presence of *X* plasmid transfection, consistent with the higher AAV2 DNA replication. We further analyzed the effects of *X* on AAV2 replication in a similar type of experiment to that of Figure 5C, shown in Figure 5F, but with an increasing transfection of the X expression plasmid, as indicated. It was found that increasing dose of *X* plasmid resulted in corresponding increasing levels of autonomous AAV2 DNA replication. This analysis confirms the importance of *X* in autonomous wild type AAV2 DNA replication.

### *X* contributes to dl78-91 DNA replication in 293 cells

**Figure 6:**
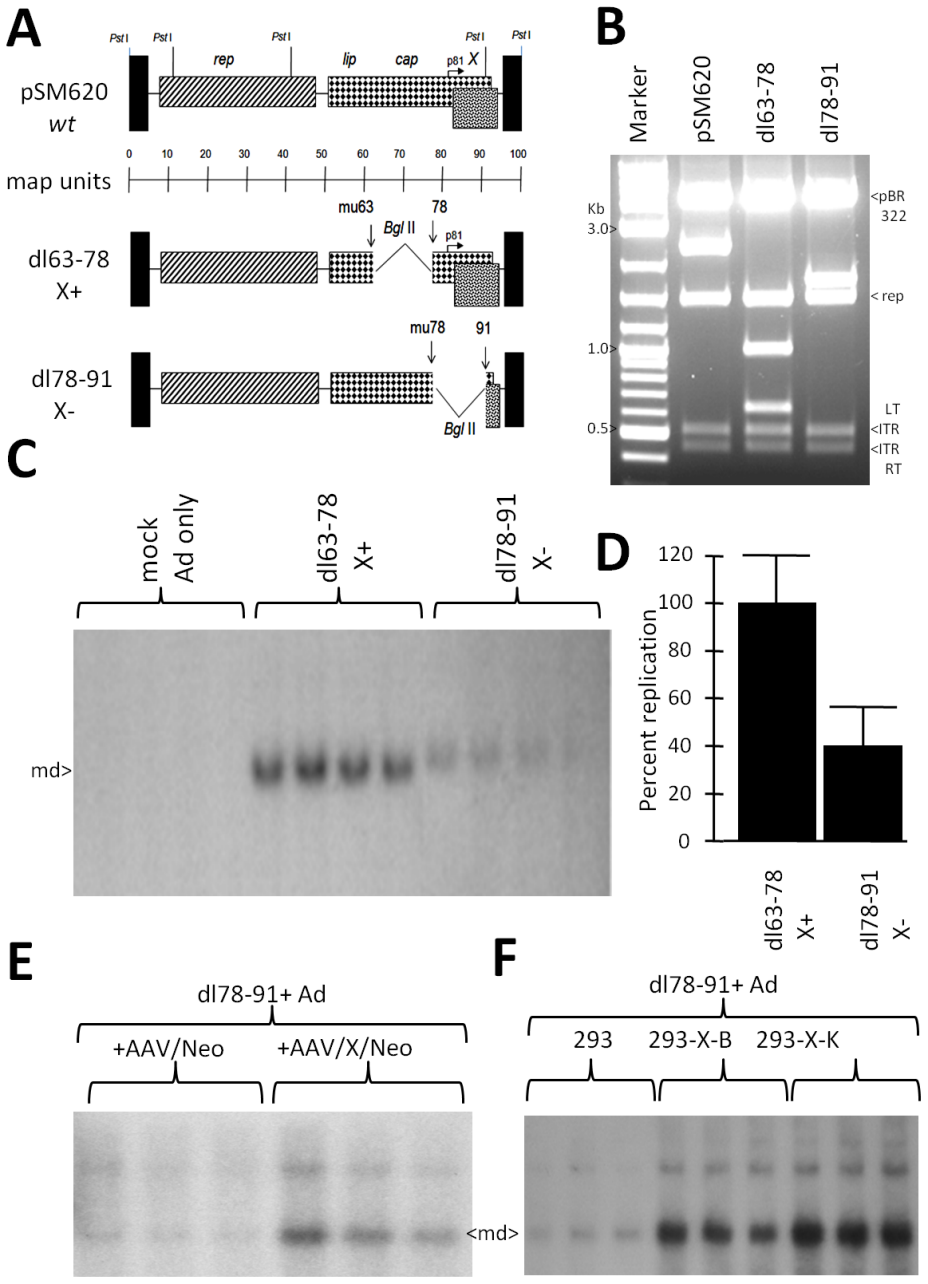
Deletion of *X* gives lower DNA replication of AAV2. **A** shows the structure of AAV2 deletion mutants dl63-78 and dl78-91, with wild type (wt) AAV2 shown at the top, including *Pst* I restriction sites. **B** shows a *Pst* I, *Bgl* II dual digestion of dl63-78 and dl78-91. **C** shows a Southern blot analysis comparison of dl63-78 and dl78-91 DNA replication in Ad5-infected 293 cells, probed with 32P-*rep* DNA, and densitometrically quantitated in **D**. Note that dl63-78 replicates approximately 2.5 fold higher than the dl78-91(*p*<0.05). **E** shows a comparison of dl78-91 DNA replication upon co-transfection with either AAV/Neo or AAV/X/Neo into Ad5-infected 293 cells (*p<*0.05). **F** shows a Southern blot analysis comparison of dl78-91 DNA replication in Ad5-infected unaltered 293 cells, 293-X-B, and 293-X-K, probed with 32P-*rep* DNA. An analysis of the level of copy numbers of *X* in these cells is shown in Figure 4 C. Note that dl78-91 replicates to higher levels in the 293 cells which contain the *X* gene (complementation) compared to unaltered 293 cells without *X* (*p*<0.05).

We next tested the involvement of *X* in the AAV2 life cycle in 293 cells using the somewhat similar assay as to that in Figure 5 using keratinocytes, however 293 cells were Ad5 infected (moi of 10) and also transfected with a deletion mutant (dl) dl63-78 or dl78-91 plasmid. The structure of these two mutants is shown in Figure 6A and an analysis of their structures by *Pst* I restriction digestion is shown in Figure 6B. DL78-91 is a deletion mutation of p81-X expression cassette within the AAV genome, as shown in Figure 6A, and as such it is *rep*+, can replicate its DNA, but can't make virus as it is also *cap*-. In comparison, dl63-78 was a slightly larger deletion than dl78-91, located just upstream of the dl78-91 deletion and leaving the p81-*X* expression cassette intact. The pSM620-derived mutant plasmid transfected 293 cells were harvested at two days post-Ad5 infection and total cellular DNA analyzed for AAV2 DNA replication by Southern blot using ^32^P-*rep* probe, shown in Figure 6C, and a densitometric quantification of the results is shown in Figure 6D. As can be seen dl63-78, with an intact *X* gene, was able to replicate at a 2.5 fold higher level than dl78-91 (*p<*0.05), again suggesting that *X* is involved in AAV2 DNA replication in 293 cells, as it was found in differentiating primary keratinocytes. We next tried to complement the defective phenotype of dl78-91 with *X*. Ad5 infected (moi of 10)-293 cells were transfected with dl78-91 plasmid plus *X* expressing plasmid, or control Neo-only plasmid. The transfected 293 cell lines were harvested at two days post-Ad5 infection and analyzed for DNA replication. Figure 6E shows a ^32^P-*rep* DNA probed Southern blot of the results, and as can be seen the level of monomer duplex (md) AAV2 dl78-91 DNA (4.1 kb) is three times higher in the presence of *X* –expressing plasmid (*p<*0.05), consistent with *X* playing a role in DNA replication. We then used the X-positive 293 cells (293-X-B and 293-X-K) as seen in Figure 4C, to confirm complementation of dl78-91 by *X* to give higher DNA replication. DL78-91 plasmid was transfected into equivalent plates (70% confluent) of unaltered 293, 293-X-B, and 293-X-K, all of which had all been infected with Ad5 (moi 10). In the resulting Southern blot notice that dl78-91 reached a higher level of DNA replication when *X* was present (5–6× higher) indicating the beneficial effects of *X* for AAV2 DNA replication (*p<*0.05).

### *X* contributes to AAV2 (pSM620) DNA replication in 293 cells

**Figure 7:**
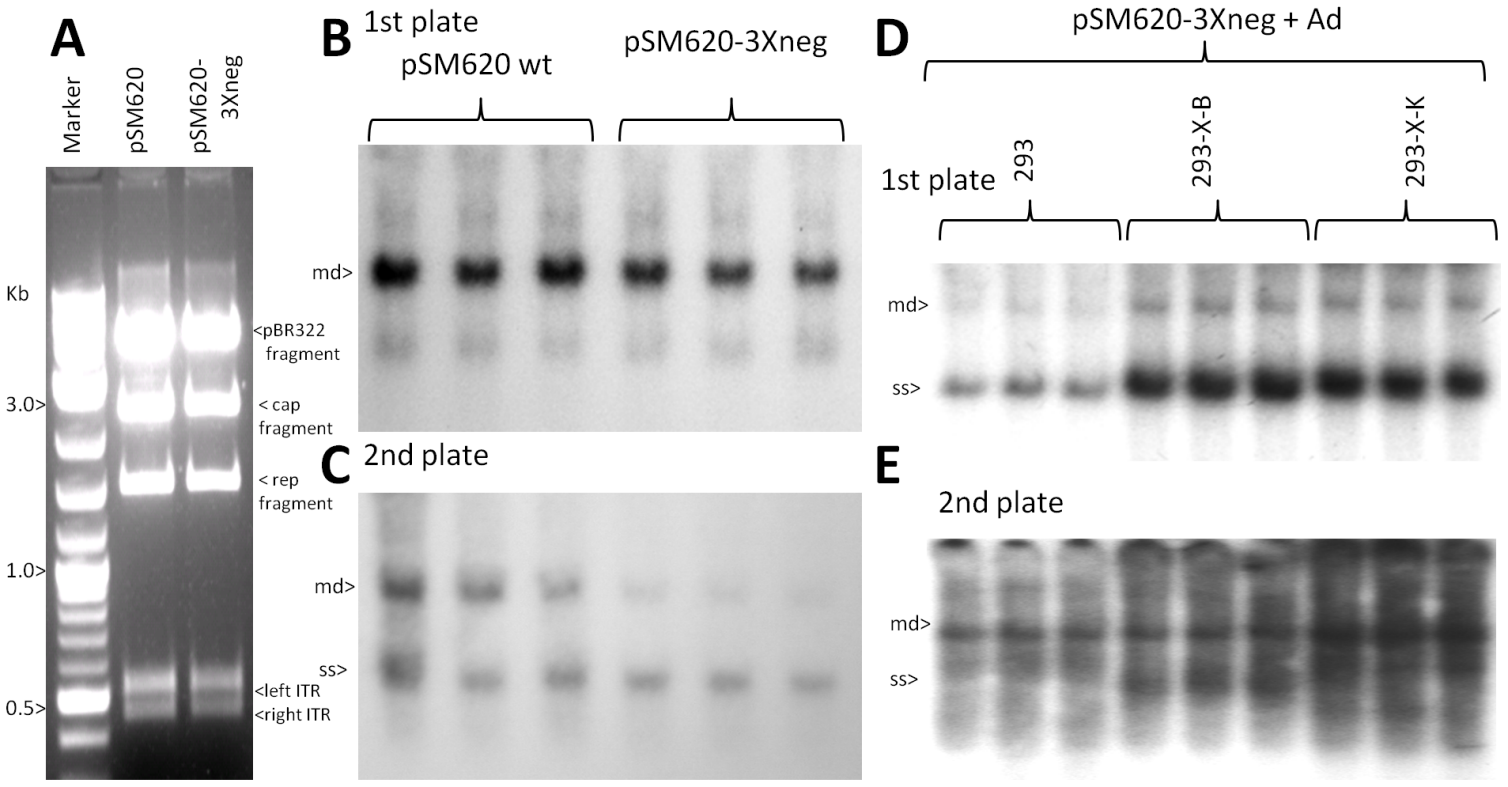
pSM620-3Xneg, without *X*, displays weaker DNA replication in Ad5-infected 293 cells. **A** shows a *Pst* I restriction digestion analysis of wt pSM620 and pSM620-3Xneg (*X-*). **B** shows the Southern blot of DNA replication, using ^32^P-*rep* pribe, of pSM620 and pSM620-3Xneg relative to each other in Ad5-infected 293 cells. Note that pSM620 replicated to a slightly higher level than pSM620-3Xneg (*p* = 0.057). **C** shows a “2^nd^ plate analysis” where equal aliquots of virus stock from plates identical to those of **B** where heated to 56°C (to kill Ad5), and then used to infect a second plate of Ad5-infected 293 cells. Shown is the Southern blot of DNA replication, using P32-*rep* probe, of pSM620 and pSM620-3Xneg replication from resulting first plate generated virus infection (*p*<0.05). **D** shows a Southern blot analysis comparison of pSM620-3Xneg replication in Ad5-infected unaltered 293 cells, 293-X-B, and 293-X-K, probed with ^32^P-*rep* DNA. Note that pSM620-3Xneg replicates to higher levels in the 293 cells which contain the *X* gene (complementation) compared to 293 cells without *X* (*p*<0.05). **E** shows another “2^nd^ plate analysis” where equal aliquots of virus stock from plates identical to those of **D**, which where heated to 56°C (to kill Ad5), and then used to infect a second plate of Ad5-infected 293 (normal) cells. Shown is the Southern blot of DNA replication, using ^32^P-*rep* probe, of pSM620-3Xneg replication from resulting first plate generated virus infection. Note that, pSM620-3Xneg replicated to higher levels in the 2^nd^ plate (*p<*0.05) due to higher levels of virus produced in the first plate.

To determine the affect of *X* within the context of the complete AAV2 genome we compared fully wild type pSM620 to pSM620-3Xneg. Figure 7A shows a *Pst* I digestion of pSM620 and pSM620-3Xneg, which can identify major deletions or rearrangements to the DNA, particularly to the ITRS. No differences were observed. Ad5 infected (moi of 10)-293 cells were then transfected with pSM620 to pSM620-3Xneg plasmid and the DNA of the transfected 293 cells harvested at two days post-Ad5 infection and analyzed for DNA replication, and equivalent plates were used to compare virion production. Figure 7B shows a ^32^P-cap DNA probed Southern blot of DNA replication, and as can be seen the level of monomer duplex (md) wt AAV2 (4.7 kb) of pSM620 was ∼33% higher than the level of AAV2-3Xneg (*p* = 0.057). Equal aliquots (300 µl) of resulting virus stock were heated to 56°C for 30 minutes (heat kill Ad5) and used to infect a second plate of Ad5-infected HEK293 cells. Figure 7C shows a ^32^P-rep (1.5 kb *Pst* I fragment) DNA probed Southern blot of the 2^nd^ plate DNA replication, and as can be seen the level of monomer duplex (md) wt AAV2 (4.7 kb) of pSM620 was ∼66% higher than the level of AAV2-3Xneg replication (*p<*0.05). This is consistent with an accumulative compounding of the weaker virion production/replication of pSM620-3Xneg in the first plate plus, again, lower DNA replication of the resulting AAV2-3Xneg in the second plate.

We again used the X-positive 293 cells (293-X-B and 293-X-K) to observe if there was any *X* complementation of pSM620-3Xneg during DNA replication. pSM620-3Xneg plasmid was transfected into equivalent plates (70% confluent) of unaltered 293, 293-X-B, and 293-X-K, all of which were infected with Ad5 (moi 10). In the resulting Southern blot (Figure 7D) notice that pSM620-3Xneg reached a higher level of DNA replication in the 293-X-B (2.9 fold higher) and 293-X-K (3.7 fold higher) cells than in the 293 cells (*p*<0.05), again verifying the contribution of *X* to AAV2 DNA replication. Equal aliquots (300 µl) of resulting virus stock were heated to 56°C for 30 minutes (heat kill Ad5) and used to infect a second plate of Ad5-infected HEK293 cells. Figure 7E shows a ^32^P-*rep* DNA probed Southern blot of the 2^nd^ plate DNA replication, and as can be seen the level of monomer duplex (md) AAV2-3Xneg (4.7 kb) DNA was 2.3 fold and 4.4 fold higher when generated by the 293-X-B and 293-X-K cells, respectively, compared to when generated from unaltered 293 cells (*p<*0.05). Thus in all assays thus far tested the lack of *X* resulted in lower DNA replication levels and production of AAV2 virus.

### *X* contributes to rAAV/eGFP DNA replication and virion production in 293 cells

**Figure 8:**
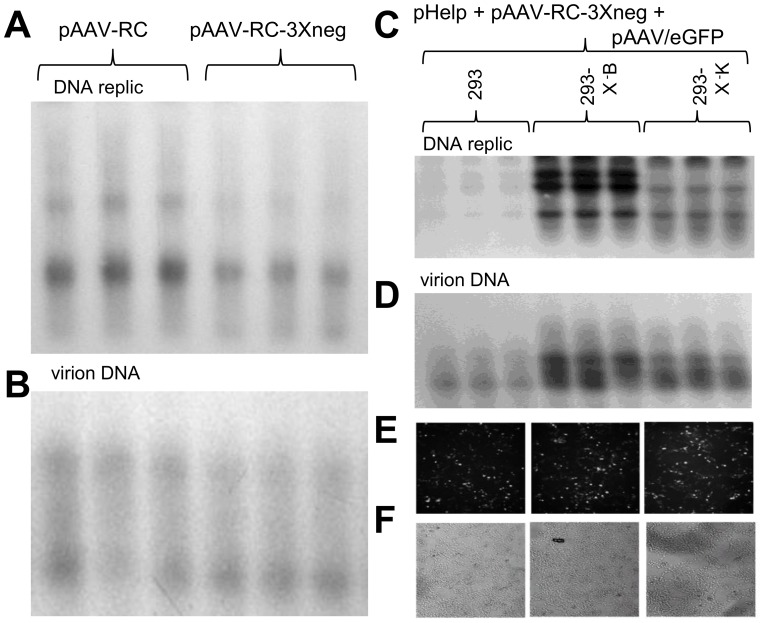
Recombinant defective (r)AAV DNA replication and virion production are lower without *X*. **A** shows the Southern blot analysis of rAAV/eGFP DNA replication, using ^32^P-*eGFP* probe, resulting from the standard 293 cell triple transfection procedure (pAAV/eGFP, pHelper, pAAV-RC) except comparing the usage of either wt pAAV-RC or pAAV-RC-3Xneg. Note that use of pAAV-RC resulted in slightly higher pAAV/eGFP DNA replication levels than when using pAAV-RC-3Xneg (*p*<0.05). **B** shows a Southern blot analysis of DNAse I resistant virion DNA (encapsidated genomes). Again note that the use of wt pAAV-RC resulted in higher rAAV/eGFP virion levels (*p*<0.05). **C** shows a Southern blot which (^32^P-eGFP probe) which compares the use of pAAV-RC-3Xneg, along with pAAV/eGFP and pHelp, to replicate AAV/eGFP DNA in unaltered 293, versus 293-X-B and 293-X-K cells, both of which contain the *X* gene. Note that higher DNA replication levels of AAV/eGFP take place in the *X-*positive 293-X-B and 293-X-K cells than normal 293 cells (*p<*0.05). **D** shows a Southern blot analysis of DNAse I resistant virion DNA (encapsidated genomes). Again note that the use of 293-X-B and 293-X-K cells, having the *X* gene, resulted in higher rAAV/eGFP virion levels (*p*<0.05). **E** shows an analysis of eGFP expression/virion infectivity in which AAV/eGFP virus, equalized for comparable titers from quantitative densitometric analysis of the virion DNA Southern blot in panel **D** was used to infect normal 293 cells and analyzed for eGFP expression at two days post-infection. Note that equal eGFP expression can be seen across all three cell infections indicating that the use of pAAV-RC-3Xneg with the 293-X-positive cell lines gave virus with comparable infectivity to the standard pAAV-RC/wt 293 cell production scheme. **F** show a white light picture of the same field depicted in **E** as a control for cell viability.

While these analyses of wild type AAV2 autonomous replication in skin and in HEK 293 cells is critically important to understand the effects of *X* within the normal AAV2 viral life cycle, most researchers want to known about *X*'s effects on recombinant (r)AAV2 DNA production, as AAV-based gene delivery is now a growing industry. To determine the affect of *X* on rAAV production the rAAV2/eGFP virus stocks were produced by the triple transfection of pAAV/eGFP, pHelper, and pAAV-RC, with the exception that were indicated pAAV-RC3Xneg was used in place of pAAV-RC. Seventy percent confluent 293 cells were transfected with those three plasmids, including the trade off of either pAAV-RC or pAAV-RC3X and analyzed for DNA replication, and equivalent plates were used to compare virion production. Figure 8A shows a ^32^P-eGFP DNA probed Southern blot of DNA replication, and as can be seen the level of monomer duplex (md) wt AAV2/eGFP (2.0 kb) was 50% higher using wt pAAV-RC than with pAAV-RC-3Xneg (*p*<0.05). Equal aliquots (300 µl) of resulting virus stock were then analyzed for DNase I-resistant encapsidated DNA (virion DNA). Figure 8B shows a ^32^P-eGFP DNA probed Southern blot of the virion DNA which was also similarly 50% higher as was the level of DNA replication (*p<*0.05).

We again used the X-positive 293 cells (293-X-B and 293-X-K) to observe if there was any form of complementation of pAAV-RC-3Xneg during rAAV DNA replication and virion production. rAAV2/eGFP virus stocks were produced by the triple transfection of pAAV/eGFP, pHelper, and pAAV-RC-3Xneg, into the 293, 293-X-B and 293-X-K cells (70% confluent). The resulting Southern blot analysis (Figure 8C) shows that pAAV/eGFP DNA replication levels were 4.2 fold and 2.3 fold higher in 293-X-B and 293-X-K cells, respectively, than in the unaltered 293 cells (*p<*0.05). Yet again this verifies the contribution of *X* to rAAV/eGFP DNA replication. Equal aliquots (300 µl) of resulting virus stock were then analyzed for DNase I-resistant encapsidated DNA (virion DNA). Figure 8D shows a ^32^P-eGFP DNA probed Southern blot of the virion DNA were 3.6 fold and 2.6 fold higher in 293-X-B and 293-X-K cells, respectively, than virus stock from the unaltered 293 cells (*p<*0.05). In summary, all these data are consistent with AAV X playing a role in AAV2 replication.

## Discussion

Although largely ignored for over the thirty years since its discovery during the first sequencing of the AAV2 genome, the *X* ORF [10], [11] is rather conspicuous by any ORF finder analysis of AAV2, being the third largest such ORF in the AAV2 genome. This study demonstrates that when the AAV2 *X* is present it increases AAV2 autonomous DNA replication (no helper) in differentiating keratinocytes, its natural host tissue, in AAV2 DNA replication in Ad5-infected 293 cells, and rAAV2/eGFP replication/virion production in HEK 293 cells with complementation by pHelper and pAAV-RC plasmids. We need to point out that X might not be directly involved in DNA replication but rather some yet to be determined supportive mechanism. It should also be noted that Buller and Rose (1978) found AAV2-induced proteins of 16 and 25 kDa in size [16] and one (or both) of these may be the *X* protein.

This study demonstrates that the AAV2 *X* gene has an effect on AAV2 biology in two different tissue culture systems (primary keratinocytes and HEK293 cells), and the replication of both the full length AAV2 genome and fully defective rAAV2/eGFP recombinant. While not commonly used for AAV study, AAV2 is able to productively replicate, without the presence of helper virus, in the skin raft culture system [12]–[15], and in this system augmentation of *X* expression by plasmid transfection gave rise six fold higher AAV2 DNA replication. Additionally, we utilized one of the standard systems for production of rAAV2 which includes the use of pHelper (containing the Adenovirus helper genes) and pAAV-RC (containing the AAV *rep* and *cap* genes) in HEK293 cells. In this system we compared pAAV-RC-3Xneg in which the *X* ORF was fully incapacitated by having the three most 5′ ATGs knocked out (pAAV-RC-3Xneg) to fully wild type pAAV-RC and found that both rAAV DNA replication and virion production were mildly inhibited by about half (statistically significant).

The level of replication boost provided by *X* appears to be most strong in differentiating keratinocytes and in 293-X-B/293-X-K cells versus normal 293 cells, yet in all cases the increase in DNA replication induced by *X* was statistically significant. Why there are differences in the strength of augmentation of the various forms of AAV2 replication assayed for is presently unclear. It is also noteworthy that in differentiating keratinocytes increased X expression resulted in higher AAV2 replication (Figure 5F). These data suggest that the level of X may be a bottleneck for AAV2 replication. As for the production of rAAV for use in gene therapy, as all of the standard production schemes include the *lip-cap* gene, thus they also contain *X* (ending at nt 4393) which is fully overlapping with *cap* (ending at nt 4407). Transcripts originating from the p81 promoter, just up-stream from *X*, were confirmed by both S1 nuclease protection and primer extension [10]. Thus *X* is an ORF expressed from a dedicated promoter, p81 [10], and this is consistent with *X* as likely being an important gene within the AAV2 life cycle.

That the *X* ORF is conserved in all AAV2 isolates looked at (Figure 1D) and considering that AAV2 is the most common AAV found in humans [17]–[19], also suggests that *X* is important. ProteinFun 2.2 analysis (Figure 2) gave us suggestions as to the function of *X* as being involved in DNA replication and transcription. Moreover, the knowledge that X has three methionine codons within the 5′ half suggests that there may be multiple X proteins produced with perhaps differing activities. Additionally, it is important to point out that an X ORF at the 3′ end of the *cap* gene is found in a least one member of every AAV clade as shown in Table 1. Thus future research will address what is the specific role of X in AAV DNA replication, and if X has effects on AAV2 transcription and on the regulation of the p81 promoter. However, here, it is exciting that *X* has been finally discovered to have an identifiable phenotype nearly fifty years after AAV's discovery [10], [11].

**Table 1:** Presence and homology of AAV2 X with that of the possible X proteins encoded by other AAV clades and types.

Clade	Representative	Identities	Positives	Gaps
Clade A	AAV1 ORFA	37/77 (48%)	46/77 (60%)	0/77 (0%)
Clade B	hu.29R	155/155 (100%)	(100%)	0/155 (0%)
Clade C	hu.11	21/42 (50%)	24/52 (57%)	0/42 (0%)
Clade D	cy.5R4 ORF B	58/97 (60%)	65/97 (67%)	8/97 (8%)
Clade D*	cy.5R4 ORF A+B	80/142 (56%)	91/142 (64%)	12/142 (8%)
Clade E	AAV8	28/66 (42%)	38/66 (58%)	1/66 (1.5%)
Clade F	AAV9	22/50 (44%)	29/50 (58%)	0/50 (0%)
AAV3	AAV3	22/40 (45%)	26/44 (59%)	2/44 (5%)
AAV5 Clade	Go.1	4/9 (44%)	5/9 (56%)	0/9 (0%)
AAV10	AAV10	25/38 (66%)	26/38 (68%)	0/38 (0%)
AAV12	AAV12	17/34 (50%)	18/34 (53%)	0/34 (0%)
Rh.39	Rh.39	48/77 (62%)	55/77 (71%)	0/77 (0%)
Hu.T88	Hu.T88	141/155 (91%)	145/155 (93%)	0/155 (0%)

Shown are the results of NCBI ProteinBLAST software analysis comparing the indicated proteins derived from ORFs in the same relative position as AAV2 X within the AAV2 genome.

*Refers to the combined amino acids of cy.5R4 X ORFs fused A before B.

## Materials and Methods

### Virus and Cells

Cloned AAV2, pSM620, titered AAV2, and adenovirus type 5 viral stocks were originally obtained from Dr. Ken Berns. pAAV-RC-3Xneg was generated from pAAV-RC (Stratagene) by GenScript with mutations dictated as in Figure 4B. pSM620-3Xneg was generated by replacing the *Bsi*W I-*Sna*B I fragment (AAV2 sequence nt 3254–4497) of pSM620 with that from pAAV-RC-3Xneg. Dl63-78 (dl, deletion) was generated by ligating the appropriate *Bgl* II-*Eco* RV fragments from ins63 (ins, insertion of *Bgl* II linker) and ins78 [1]. Dl78-91 was generated by ligating the appropriate *Bgl* II-*Eco* RV fragments from ins78 and ins91 [1]. AAV/eGFP was generated by ligating the eGFP coding sequence into the *Xho* I site just behind the CMV promoter in dl3-97/CMV. pCI-X-Neo was generated by cloning the X open reading frame into the *Xho* I and *Sal* I sites of pCI-Neo. Primary human foreskin keratinocytes were obtained from Clonetics. J2 fibroblasts and HEK293, hereafter called 293 cells (Hermonat *et al.*, 1997) cells have been described previously [11]–[13]. Primary human foreskin keratinocytes (PHFK)(Clonetics) were maintained in keratinocyte SFM medium from GibcoBRL±Life Technologies (Cat. No. 10724-011). Epithelial organotypic rafts were maintained in E medium, which has been described previously [11]–[13]. 293 cells were maintained in Dulbecco's modification of Eagle's medium with 7% fetal bovine serum and antibiotics.

### Transfection and generation of epithelial organotypic rafts

Primary human foreskin keratinocytes (PHFK) were transfected with AAV/*X*/Neo or AAV/Neo plasmids (5 µg each, or as indicated) into 3×10^5^ PHFK using Fugene6 per manufacturer's instructions (day 0). Transfection efficiency by this technique is 45% [13]. The next day the cultures were infected with a multiplicity of infection (moi) of 100 AAV2 virus (day1). The following day the cells were trypsinized and epithelial raft tissues were generated as described previously [11]–[13], with the exception that no protein kinase C inducers, such as TPA, were added to the culture medium. Briefly, 3×10^5^ of the transfected/infected PHFK were plated onto collagen disks containing J2 fibroblast cells submerged in E medium and the cells were allowed to adhere for 2 h and then the raft lifted to the air±liquid interface (day 2). The raised raft cultures were allowed to stratify and differentiate as previously described [11]–[13] and the experiment is depicted in Figure 5B.

### Analysis of AAV DNA replication in epithelial organotypic rafts by Southern blot

Total DNA was isolated from the raft tissues. The raft tissue was minced and placed in 500 µl of lysis buffer [5 mM Tris/HCl (pH 7.4), 5 mM EDTA, 0.25 mg/ml proteinase K]. After tissue was digested, the solution was phenol extracted and ethanol precipitated to purify total cellular DNA. For the measurement of AAV progeny formation by second plate amplification assay, after 36 h Hirt DNA was isolated from these second plate amplifications as previously described [11]–[13].

### Analysis of AAV2 rep and cellular TF_II_B mRNA expression in epithelial organotypic rafts by RT-PCR

Total RNA was isolated from rafts on day 5 using Trizol reagent (Invitrogen, Carlsbad, CA), according to the manufacturer protocol and treated with 5 U/Ag of RNase-free DNase I at 37 -C for 2 h. Messenger polyA RNA then was isolated using the Oligotex mRNA Mini Kit (QIAGEN Inc. Valencia, CA) according to the supplier's instruction. The first-strand cDNA synthesis was performed at 37 -C for 1 h in a final volume of 25 Al reaction buffer (1 Ag mRNA; 50 mM Tris–HCl, pH8.3; 75 mM KCl; 3 mM MgCl2; 10 mM DTT; 0.5 Ag oligo(dT)15; 0.5 mM each of the four dNTPs; 30 U of RNasin and 200 U of M-MLV Reverse Transcriptase RNase H Minus (Promega Co., Madison, WI)). PCR amplification (32 cycles) of the cDNA was performed in a 100-Al reaction volume which contained 2.5 U Taq DNA polymerase; 10 mM Tris–HCl, pH8.3; 50 mM KCl; 2 mM MgCl2; 0.2 mM each of the four dNTPs; 1 AM of each upstream and downstream primer specific for the cDNA template and 10 Al cDNA templates. The primer set used for AAV rep was 5V-TGAAGCGGGAGGTTTGAACG-3V and 5V-TCCATATTAGTCCACGCC-3V, which targeted amplification of the AAV sequences from nt 291 to 821. The TF_II_B (housekeeping gene) was also analyzed in each RT-PCR mix. The products were size separated by agarose gel electrophoresis, stained with ethidium bromide and photographed.

### Analysis of AAV2 DNA replication in 293 cells

293 (6 cm plates) cells at 70% confluence were transfected (Fugene 6, Roche) with 3 µg of the indicated plasmid. Fugene 6 gives high efficiency transfection, yet relatively low altered endogenous cellular gene expression [20]. Then the 293 cells were infected with Ad5 (moi 10) for helper function the cells were harvested at 2 days post-transfection. When the 293 cells were transfected with pHelper (Ad5 helper genes) and pAAV-RC (AAV *rep* and *cap*) plasmids, the cells were harvested on day 5. Cells were lysed with 1.5 ml of 1% SDS, 7.2 pH Tris-HCL, 5 mM EDTA, and Pronase K and incubated overnight. The total cellular DNA was then drawn though a 20 gauge needle ten times (to make less viscous), phenol extracted, ethanol precipitated twice, and 10 µgs of DNA were agarose gel electrophoresed, Southern blotted and probed with the indicated ^32^P-labeled DNA probe. When, 2^nd^ plate virus production analysis was done, cells/medium were freeze-thawed three times, heated to 56°C for 30 minutes, and 300 µls (or as indicated, from a total of 5 ml) was then used to infect a second plate of 293 (6 cm plates) cells at 70% confluence which were infected with Ad5 (moi 10). At two days post-infection total cellular DNA was isolated and analyzed by Southern blot as just described. After autoradiography densitometric analysis was carried out using the Alpha Imager 2000 with resident software (Alpha Innotech Corporation, San Leandro, CA). P value, were determined using the Microsoft Excel software for the 2-tailed Student Ttest.

### Virion DNA analysis

Six cm plates of transfected 293 cells were freeze-thawed three times, cellular debris pelleted by centrifugation at 7,000 rpm for 25 minutes, and the supernatant pushed through a 0.22 µm filter. Three hundred µl of virus stock was treated with 20 units DNase I for 30 minutes at 37°C. After heating the sample for 10 minutes at 100°C, the sample was digested with proteinase K (0.2 µg/ml) for 4 hrs, then phenol extracted and ethanol precipitated (with addition of 10 µg tRNA). The resulting DNA was then agarose gel electrophoresed, Southern blotted and probed with ^32^P-eGFP DNA when analyzing for rAAV production or with ^32^P-pSM620 DNA, when analyzing for wt AAV production.

### Infectivity assay

AAV/eGFP virus stock was equalized according to the relative titer determined by the densitometric analysis of DNase I-resistant virion DNA and 100–400 µls (equalized for amount of virus) of AAV/eGFP virus stock were used to infect 70% confluent plates of 293 cells. AAV/eGFP transduction was measured by eGFP fluorescence at 48 hours post-infection.

### Statistics

Parameters were analyzed with statistics software SPSS 16.0 by nonparametric ANOVA test. If differences were detected between means, Newman-Keuls test was used for multiple comparisons. Difference were considered as significant if *P*<0.05.

### Western blot analysis of X protein

Anti-98 rabbit polyclonal antibody was generated by GenScript against the peptide amino-TPRDRSAWRSSGSC-carboxy, representing X sequences from aa 98–111. Total protein was extracted from the 293 cells in the CelLytic M mammalian Cell Lysis/Extraction reagent (SIGMA). Protein concentration was determined using the protein assay dye reagent (Bio-RAD) and were normalized for equal loading. After separating on 10% SDS-PAGE gels, protein was transferred to Immun-Blot PVDF membranes. The membranes were then blocked for 1 hour at room temperature with 5% nonfat milk in 1× TBST buffer (10 mM Tris-Cl (pH 7.5), 150 mM NaCl, 0.1% Tween 20). Followed a brief rinse, membranes were incubated with polyclonal anti-98-X-horseradish-peroxidase (HRP)-conjugated antibody (1∶500 dilution, Sigma-Aldrich) at 4°C overnight. Washes in 1× TBST buffer were performed between incubations for three times. Blots were developed with Pierce ECL system (Thermo-Fisher Scientific). Probe detection of β-actin was carried out as control.

## References

[1] Hermonat PL, Labow MA, Wright R, Berns KI, Muzyczka N (1984) Genetics of adeno-associated virus: isolation and preliminary characterization of mutants in adeno-associated virus type 2. J Virol 51: 329–339. 6086948 10.1128/jvi.51.2.329-339.1984 PMC254442

[2] Hermonat PL, Muzyczka N (1984) Use of adeno-associated virus as a mammalian DNA cloning vector: transduction of neomycin resistance into mammalian tissue culture cells. Proc Natl Acad Sci USA 81: 6466–6470. 6093102 10.1073/pnas.81.20.6466 PMC391945

[3] Hermonat PL (1984) Genetic analysis and utilization of adeno-associated virus as a mammalian cloning vector. Univ FL Dissertation, Available: http://archive.org/details/geneticanalysisu00herm.

[4] Hermonat PL (2014) The first adeno-associated virus gene transfer experiment, 1983. *In press* Human Gene Therapy 10.1089/hum.2014.047 24950088

[5] You CX, Shi M, Liu Y, Cao M, Luo RC, et al (2012) AAV2/IL-12 gene delivery into dendritic cells (DC) enhances CTL stimulation above other IL-12 applications: evidence for IL-12 intracrine activity in DC. Oncoimmunology 1: 847–855. 23162752 10.4161/onci.20504 PMC3489740

[6] Zhu H, Cao M, Straub KD, Hermonat PL (2013) Systemic delivery of thiol-specific antioxidant hPRDX6 gene by AAV2/8 inhibits atherogenesis in LDLR KO mice on HCD. J Genet Syndr Gene Ther 4: 135.

[7] Buchlis G, Podsakoff GM, Radu A, Hawk SM, Flake AW, et al (2012) Factor IX expression in skeletal muscle of a severe hemophilia B patient 10 years after AAV-mediated gene transfer. Blood 119: 3038–3041. 22271447 10.1182/blood-2011-09-382317 PMC3321866

[8] Tratschin JD, Miller IL, Carter BJ (1984) Genetic analysis of adeno-associated virus: properties of deletion mutants constructed in vitro and evidence for an adeno-associated virus replication function. J Virol 51: 611–619. 6088786 10.1128/jvi.51.3.611-619.1984 PMC255808

[9] Sonntag F, Schmidt K, Kleinschmidt JA (2010) A viral assembly factor promotes AAV2 capsid formation in the nucleolus. Proc Natl Acad Sci USA 107: 10220–10225. 20479244 10.1073/pnas.1001673107 PMC2890453

[10] Hermonat PL, Santin AD, DeRijcke M, De Greves J, Bishop B, et al (1999) Chromosomal latency and expression at map unit 96 of a wild-type plus adeno-associated virus vector(AAV)/Neo vector and identification of p81, a new AAV transcriptional promoter. J Human Virol 2: 359–368. 10774553

[11] Srivastava A, Lusby EW, Berns KI (1983) Nucleotide sequence and organization of the adeno-associated virus 2 genome. J Virol 45: 555–564. 6300419 10.1128/jvi.45.2.555-564.1983 PMC256449

[12] Meyers C, Mane M, Kokorina N, Alam S, Hermonat PL (2000) Ubiquitous adeno-associated virus type 2 replicates in a model of normal skin. Virology 272: 338–346. 10873777 10.1006/viro.2000.0385

[13] Meyers C, Alam S, Mane M, Hermonat PL (2001) Altered biology of adeno-associated virus type 2 and human papillomavirus during dual infection of natural host tissue. Virology 287: 30–39. 11504539 10.1006/viro.2001.0968

[14] You H, Liu Y, Prasad CP, Agrawal N, Zhang D, et al (2006) Multiple human papillomavirus genes affect the adeno-associated virus life cycle. Virology 344: 532–40. 16203022 10.1016/j.virol.2005.08.039

[15] Kang BY, You H, Bandyopadhyay S, Agrawal N, Melchert RB, et al (2009) Cervical cancer isolate PT3, super-permissive for adeno-associated virus replication, over-expresses DNA polymerase delta, PCNA, RFC and RPA. BMC Microbiology 9: 79. 19389243 10.1186/1471-2180-9-79 PMC2685399

[16] Buller RM, Rose JA (1978) Characterization of adeno-associated virus-induced polypeptides in KB cells. J Virol 25: 331–338. 621779 10.1128/jvi.25.1.331-338.1978 PMC353931

[17] Blacklow NR, Hoggan MD, Rowe WP (1968) Serologic evidence for human infection with adenovirus-associated viruses. J Natl Cancer Inst 40: 319–327. 4295610

[18] Blacklow NR, Hoggan MD, Sereno MS, Brandt CD, Kim HW, et al (1971) A seroepidemiologic study of adenovirus-associated virus infection in infants and children. Am J Epidemiol 94: 359–366. 4329327 10.1093/oxfordjournals.aje.a121331

[19] Chirmule N, Propert K, Magosin S, Qian Y, Qian R, et al (1999) Immune responses to adenovirus and adeno-associated virus in humans. Gene Ther 6: 1574–1583. 10490767 10.1038/sj.gt.3300994

[20] Nagy V, Watzele M (2006) FuGENE 6 transfection reagent: minimizing reagent-dependent side effects as analyzed by gene-expression profiling and cytotoxicity assays. Nature Methods - 3

